# Discovery of serum APOD as an early sarcopenia biomarker in older adults with low muscle mass: a cross-sectional proteomic and transcriptomic investigation

**DOI:** 10.7717/peerj.21058

**Published:** 2026-04-14

**Authors:** Jingqiong Wu, Qinghua He, Liping Huang

**Affiliations:** 1 Department of Physical Education, Guangxi Medical University, Nanning, Guangxi, China; 2 School of Physical Education and Health Science, Guangxi Minzu University, Nanning, Guangxi, China; 3 Sports and Health College, Tianjin Medical University, Tianjin, China

**Keywords:** Sarcopenia, Serum proteomics, Function information, APOD, Diagnostic value

## Abstract

**Background:**

Sarcopenia is the progressive, widespread loss of skeletal muscle mass, strength, and function, which happens as a result of aging. This study aims to analyze the alterations in serum protein profiles and explore a biomarker of diagnostic significance for early sarcopenia patients *via* a cross-sectional study.

**Methods:**

In this cross-sectional study, serum samples from 50 older adults (25 with early sarcopenia and 25 healthy controls) were analyzed. In the discovery cohort (10 per group), proteomic sequencing was performed to identify differentially expressed proteins (DEPs). Candidate biomarker were subsequently validated using enzyme-linked immunosorbent assay (ELISA) in an independent validation cohort (15 per group). Furthermore, bioinformatics analyses, including functional enrichment and diagnostic evaluation, were conducted to elucidate the potential role of the identified targets.

**Results:**

Untargeted proteomic profiling of the discovery cohort identified 88 significantly dysregulated serum proteins in early sarcopenia, which were bioinformatically enriched in processes related to stress response and metabolic imbalance. To prioritize candidate biomarkers, the corresponding genes were cross-referenced with public transcriptomic data. This integrative analysis highlighted apolipoprotein D (*APOD*) as a leading candidate gene, demonstrating concurrent upregulation at both the proteomic and transcriptomic levels and exhibiting strong diagnostic potential (area under the curve (AUC) > 0.7). The specific elevation of serum APOD protein was subsequently confirmed through orthogonal validation *via* ELISA in an independent cohort, where its levels were significantly higher in patients with early sarcopenia compared to healthy controls. Furthermore, gene set enrichment analysis implicated *APOD* in the modulation of key pathways, including the activation of the PI3K-Akt signaling pathway and the suppression of pathways related to cardiovascular function, suggesting its potential role in the metabolic dysregulation characteristic of early muscle decline.

**Conclusion:**

This study identifies and validates serum APOD as a biomarker associated with early sarcopenia, characterized by significantly elevated levels and demonstrating promising discriminative capacity at the group level between affected individuals and healthy controls.

## Introduction

Sarcopenia is a condition characterized by the progressive loss of muscle mass, strength, and function that occurs with aging (Fielding et al., 2011). It is a prevalent condition among older adults and is associated with various negative health outcomes, such as an increased risk of falls, fractures, decreased quality of life, and even mortality (Cruz-Jentoft & Sayer, 2019). According to a recent survey, the frequency of sarcopenia among men and women living in the community was 11% and 9%, respectively, but as high as 51% and 31% in nursing facilities (Papadopoulou et al., 2020). In recent years, there has been growing recognition of the importance of identifying and treating sarcopenia. Early intervention is key to preventing or slowing down the progression of sarcopenia (Bhattacharya et al., 2022). Preventing and managing sarcopenia involves adopting a comprehensive approach that includes regular exercise, adequate nutrition, and lifestyle modifications (Phu, Boersma & Duque, 2015; Robinson et al., 2018; Liu et al., 2024). Resistance training, such as weight lifting, is particularly effective in promoting muscle growth and maintaining strength (Giallauria et al., 2016). Consuming a balanced diet rich in protein, vitamins, and minerals is also crucial for maintaining muscle mass and function (Liu, Zhang & Li, 2023). Moreover, it is crucial to raise awareness among healthcare professionals and the general population about sarcopenia’s impact and the available strategies for its management.

Sarcopenia may arise and progress due to multiple mechanisms (Kim et al., 2021), such as dysfunction of satellite cells, imbalance in protein turnover, increased reactive oxygen species and inflammation, dysfunction of mitochondria, and so forth. Additionally, certain chronic diseases such as diabetes, cancer, and heart failure can further contribute to the development and progression of sarcopenia (Curcio et al., 2020; Bossi et al., 2021; Izzo et al., 2021). Moreover, with the growing worldwide older population, sarcopenia has become a serious public health issue, inflicting a considerable social and economic burden. As a result, early detection and diagnosis of sarcopenia in community settings or nursing homes should be enhanced. Sarcopenia diagnostic criteria include assessments of muscular strength, quantity, quality, and physical performance (Cruz-Jentoft et al., 2019a). The most common technique for determining muscular strength is grip strength testing (Roberts et al., 2011), which the EWGSOP2 accepted as a standard for determining sarcopenia (Cruz-Jentoft et al., 2019b). Isokinetic testing, which is used to measure the strength of knee muscles and hamstrings, has also been used to diagnose sarcopenia (Hartmann et al., 2009). Furthermore, dual X-ray absorptiometry, bioelectrical impedance analysis, computed tomography, and magnetic resonance imaging are all common methods for measuring muscle mass (Buckinx et al., 2018). However, these diagnostic methods have disadvantages, such as ambiguous cutoff points and a lack of association between measured muscle mass and negative health effects.

Currently, proteomics has become extensively utilized in investigations seeking diagnostic markers for numerous diseases. Manousopoulou et al. (2019) have identified 21 DEPs as serological markers of endometriosis *via* proteomics analysis and comprehensive bioinformatics analysis. In a previous study, two proteins with diagnostic value for sarcopenia were identified through analysis of the serum proteomic profile of older adults with sarcopenia characterized by low grip strength (Wu et al., 2022). Accordingly, utilizing bioinformatics tools to thoroughly analyze proteomic data from patients with sarcopenia presents numerous advantages in the search for effective diagnostic biomarkers. Thus, this study aims to apply serum proteomics data in combination with bioinformatics tools in order to identify potential serological markers of early sarcopenia patients with low muscle mass.

## Subjects and methods

### Study participants

Guided by the 2018 European consensus diagnostic process and the diagnostic criteria of the Asian Working Group for Sarcopenia (AWGS), this cross-sectional study enrolled a total of 50 participants (aged >60 years) from the Tianjin Institute of Physical Education Teaching Experiment Training Center between December 2019 and December 2020. The cohort comprised 25 older adults with early sarcopenia (characterized by low muscle mass but normal handgrip strength) and 25 healthy controls (with normal muscle mass and handgrip strength). Following a comprehensive diagnostic assessment, blood samples were collected from all participants for subsequent proteomic or enzyme-linked immunosorbent assay (ELISA) analysis. A nested two-phase design was employed to establish a biomarker discovery and validation pipeline. First, a discovery cohort comprising 10 randomly selected participants from each group (10 early sarcopenia and 10 controls) underwent proteomic profiling to identify differentially expressed proteins (Table 1). Subsequently, the candidate biomarker emerging from the discovery phase were subjected to quantitative verification using ELISA in the remaining participants, who constituted an independent validation cohort (15 early sarcopenia and 15 controls) (Table 2). This study was conducted in accordance with the Declaration of Helsinki and was approved by the Institutional Ethics Committee of Tianjin University of Sport (2019-12-01). Written informed consent was obtained from all participants before their enrollment. Subject selection adhered to principles designed to minimize bias in allocation and followed a rigorous sampling protocol. Furthermore, to ensure the accuracy and reliability of all measurements, every instrument was calibrated before use.

**Table 1:** The clinical characteristics of sarcopenia patients and healthy control in the discovery cohort.

Clinical characteristics	Sarcopenia patients (*N* = 10)	Healthy (*N* = 10)	*p*
Age (mean ± SD)	67.80 ± 5.33	67.50 ± 4.99	0.898
Female, *N* (%)	4 (27.67%)	5 (33.33%)	
SMI (kg/m^2^)	5.87 ± 0.55	6.82 ± 0.72	0.004
6-Min walk test (m)	418.37 ± 69.33	438.34 ± 41.41	0.444
Handgrip strength (Kg)	27.97 ± 9.91	27.92 ± 7.63	0.99

**Note:**

SMI, Skeletal muscle index.

**Table 2:** The clinical characteristics of sarcopenia patients and healthy control in the validation cohort.

Clinical characteristics	Sarcopenia patients (*N* = 15)	Healthy (*N* = 15)	*p*
Age (mean ± SD)	68 ± 5.33	69.73 ± 4.99	0.366
Female, *N* (%)	8 (53.33%)	8 (53.33%)	
6-Min walk test (m)	415.13 ± 44.12	453.00 ± 50.58	0.037
SMI (kg/m^2^)	4.56 ± 0.68	7.21 ± 0.91	<0.0001
Handgrip strength (Kg)	39.98 ± 7.17	45.26 ± 14.92	0.227

**Note:**

SMI, Skeletal muscle index.

Additionally, datasets GSE165630, GSE111006, GSE111010, and GSE111016 were retrieved from the Gene Expression Omnibus (GEO) database, accessible at https://www.ncbi.nlm.nih.gov/geo/. The GSE165630 dataset comprised five older adults who were sedentary and nine individuals who had undergone training. In contrast, the GSE111006 dataset featured 12 subjects diagnosed with sarcopenia alongside 28 healthy controls. The GSE111010 dataset included 25 sarcopenic subjects together with 14 healthy individuals. Finally, the GSE111016 dataset encompassed 20 subjects with sarcopenia and 20 healthy controls.

### Patient selection criteria

Exclusion criteria were as described in the study subject section of the previous article (Wu et al., 2022).

Before inclusion, all subjects underwent a standardized battery of assessments. These included bioelectrical impedance analysis (BIA) for body composition, handgrip strength test using a dynamometer, and the 6-minute walk test (6MWT) to evaluate physical capacity.

The skeletal muscle index (SMI) was subsequently calculated for each individual using the following established formula (Moon et al., 2018): SMI (kg/m^2^) = Appendicular skeletal muscle mass (ASM) (kg)/height (Ht)^2^ (m) (Moon et al., 2018).

Based on the diagnostic criteria, participants were categorized into two distinct groups: Healthy control group (*N* = 25): age ≥ 60; handgrip strength: male ≥ 26 kg, female ≥ 18 kg; SMI: male > 7.0 (kg/m^2^), female > 5.7 (kg/m^2^). Early sarcopenia group (*N* = 25): age ≥ 60, handgrip strength: male ≥ 26 kg, female ≥ 18 kg; SMI: male < 7.0 (kg/m^2^), female < 5.7 (kg/m^2^).

### Collection of serum samples and preparation of proteomic samples

Fasting venous blood samples (6–8 mL) were collected from all participants, including healthy controls and individuals with early sarcopenia. After collection, the blood was allowed to clot at 37 °C for 30 min and then centrifuged at 3,000 g and 4 °C for 10 min. The resulting serum supernatant was aliquoted and stored at −80 °C until further analysis. For the subsequent proteomic sequencing, serum aliquots were strategically pooled to form analytical samples. Specifically, samples from the early sarcopenia group (*N* = 10) were combined into six sequencing samples (labeled Q1–Q6): four from individual donors, and two from pools of two or four donors each. Similarly, samples from the healthy control group (*N* = 10) were pooled in pairs, resulting in five sequencing samples (labeled N1–N5).

The Bradford assay was applied to determine the concentration of serum protein in each serum sample. Next, the serum proteins were extracted using SDT lysate (4% SDS, 150 mmol/L TRIS-HCl, 100 mmol/L DTT), and equal amounts of protein were mixed from each sample to create a pool sample for the construction of spectral libraries. SDS-PAGE electrophoresis was employed to assess the uniformity of proteins. Following that, serum proteins were enzymolyzed according to the standard FASP technique, and the peptide concentration was determined at OD280. After mixing all peptides with a 2 g indexed retention time (iRT) reference peptide, DIA-MS analysis was performed. The peptides in the pool sample were fractionated using HpRP, and all fractions were collected. Following that, 2 g of peptide from each fraction was transferred and blended with an appropriate amount of iRT standard peptide (in a volume proportion of 1:3). The resulting mixture was then analyzed using DDA-MS to build a library.

### MS method

A Thermo Scientific Q Exactive HF-X mass spectrometer connected to an Easy nLC 1200 chromatography system (Thermo Scientific, Waltham, MA, USA) for DDA and DIA analysis. For DDA analysis, the 1.5 μg peptide was injected into an EASY-Spray TM C18 Trap column and subjected to EASY Spray TM C18 LC analytical column for a linear gradient of buffer (84% acetonitrile and 0.1% formic acid) at a flow of 250 nL/min for 120 min. The detection mode was positive ion. Scan range: 300–1,800 m/z; MS1 spectral resolution: 60,000(@m/z200); AGC target: 3e6; maximum IT: 25 ms; dynamic exclusion: 30.0 s. Each full MS-SIM scan followed 20 ddMS2 scans. MS2 spectral resolution: 15,000; AGC target: 5e4; maximum IT: 25 ms: normalized collision: 30 eV.

For the DIA-MS analysis, the peptides from each sample were analyzed using LC-MS/MS in the DIA mode. MS was operated in DIA data acquisition mode, setting 30 DIA acquisition windows. Main parameter settings: Detection mode: positive ion; MS scan range: 350–1,800 m/z; mass spectrum: 12,0000(@m/z200); AGC target: 3e6; maximum IT: 50 ms; profile mode; DIA scans resolution: 15,000; max IT auto; normalized collision energy: 30 eV. The data analysis referenced the previous articles (Wu et al., 2022).

### Differential expression analysis

The differential expression analysis was conducted using the R language “limma” package (Rivera, Aposhian & Fernando, 1989). Differentially expressed proteins (DEPs) and genes (DEGs) between two groups were screened by |Log_2_FC| > 0.5 and *p* < 0.05.

### Function enrichment analysis

The DEPs to conduct the Gene Ontology (GO) terms and Kyoto Encyclopedia of Genes and Genomes (KEGG) enrichment analyses based on the Annotation, Visualization and Integrated Discovery (DAVID, https://davidbioinformatics.nih.gov/) database. The DEGs were used to conduct the GO and KEGG enrichment analysis using the R language “clusterProfiler” (Yu et al., 2012). The significantly enriched pathways were identified by *p* < 0.05.

### Construction of protein-protein interaction network

The GeneMANIA (http://www.genemania.org) was applied to comprehend the intricate interactions between proteins and assess the potential underlying pathways. The GeneMANIA database serves to generate hypotheses about gene function, analyze gene lists, and prioritize genes for functional analysis. By employing a query gene list, GeneMANIA utilizes vast amounts of genomics and proteomics data to uncover genes that exhibit similar functionalities. In this mode, it weights each functional genomic dataset according to the queried predictive value.

### ELISA

Whole blood samples were collected using clean test tubes and allowed to clot at room temperature for 30 min. The clotted samples were then centrifuged at 2,000×*g* for 20 min to obtain serum. Serum levels of apolipoprotein D (APOD) were quantified using a commercially available human APOD-specific ELISA kit (CSB-EL001935HU; CUSABIO, Wuhan, China) according to the manufacturer’s instructions. Absorbance was measured at a wavelength of 450 nm using a microplate reader.

## Results

### Quality control of protein quantification

**Figure 1:**
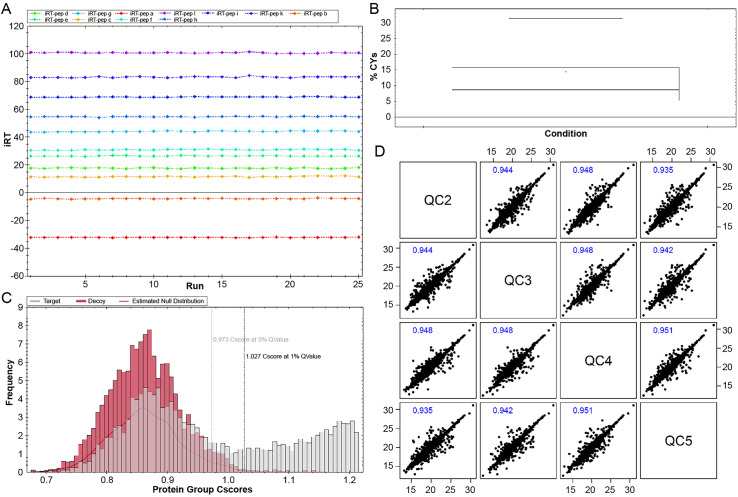
Quality control (QC) of protein quantitative. (A) The elution time of internal standard corrected peptides (iRT), the vertical axis is the retention time, and the horizontal axis is the upper machine sequence. (B) FDR distribution map. Cscore: equivalent to protein reliability score, the higher the score, the greater the reliability. Horizontal axis: Cscore value of protein; Vertical axis: the number of proteins under a certain Cscore score. Black dotted line: 1% Q-value (equivalent to 1% FDR) standard line, the higher the Cscore at the standard line, and the higher the reliability. (C) The CV values of QC, and the vertical axis was the coefficient of variation (CV%) of each sample. (D) Correlation analysis of QC samples. abscissa and ordinate were the logarithmic values of marked intensity values, respectively, and general correlation coefficients greater than 0.9 indicated good correlation.

In the DIA-seq data, there were at least five data collection points for each chromatographic peak, indicating that a sufficient density of data could be acquired in the HPLC-MS/MS data for accurate integral quantification of peptide chromatographic peaks. Internal standard corrected peptides were all detected, and retention times were generally stable (Fig. 1A). QC based on the target decoy Library search strategy was the method used when conducting the DIA data analysis. The reliability of results was high at the FDR threshold of 0.01 (Fig. 1B). Moreover, QC samples were inserted at regular intervals within the sample cohort to evaluate data consistency throughout the experiment. The analysis revealed a small intraclass coefficient of variation (CV) (Fig. 1C) and a correlation coefficient between QC samples close to 1. These results indicate that the experimental system was stable.

### Identification of early sarcopenia-associated proteins and their functional information

**Figure 2:**
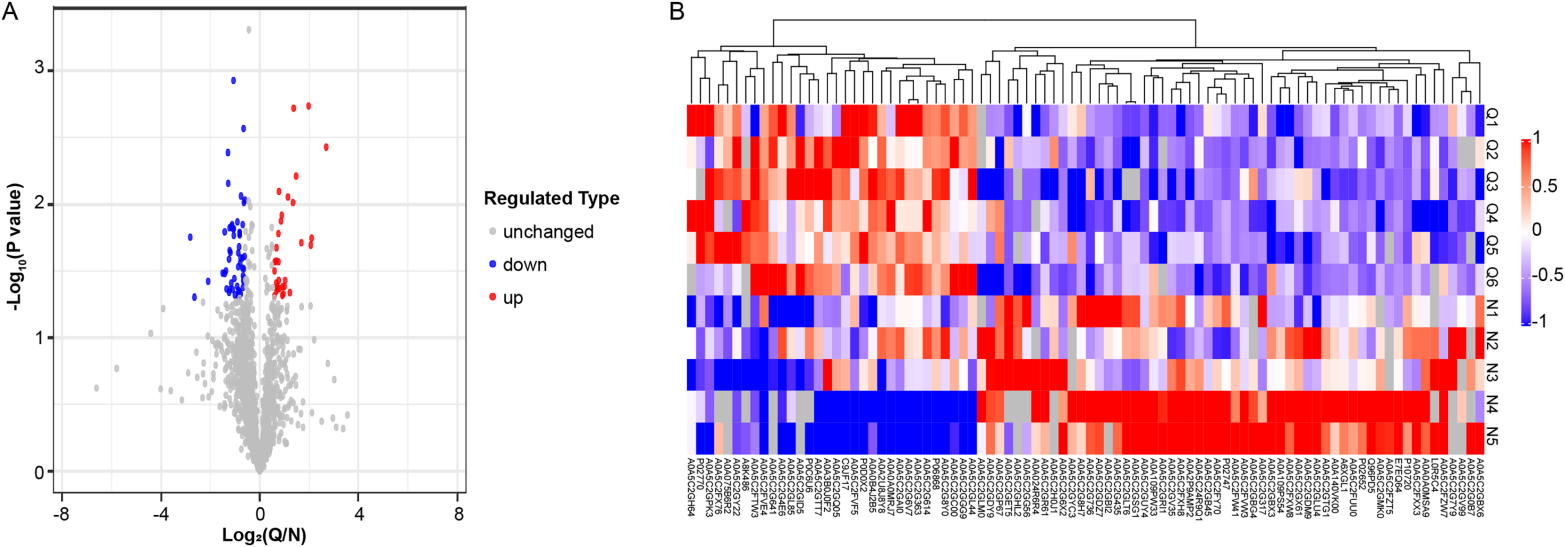
Identification of sarcopenia associated proteins. (A, B) Differentially expressed proteins (DEPs) between sarcopenia and healthy group in the discovery cohort.

In the discovery cohort (DIA-seq data), compared to the healthy group, a total of 32 proteins were significantly up-regulated, and 56 proteins were down-regulated in the early sarcopenia group (Figs. 2A, 2B). Subsequently, the coding genes corresponding to these 88 DEPs were analyzed using the UniProt (https://www.uniprot.org/) database, which resulted in 17 coding genes, including eight up-regulated and nine down-regulated genes (Table S1).

**Figure 3:**
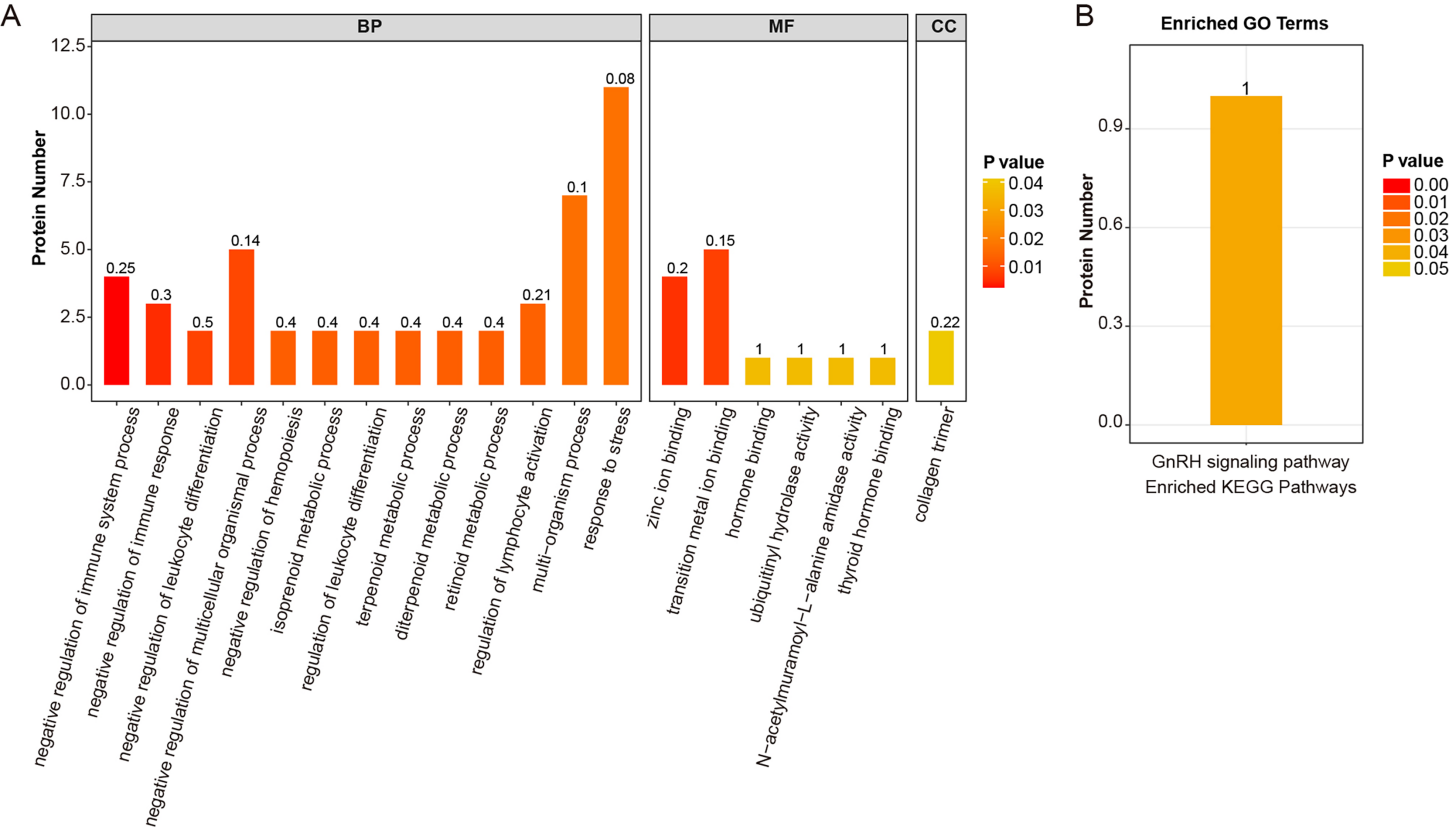
The function information of sarcopenia associated proteins. (A) The top 20 significantly enriched pathways in GO term. (B) The DEPs between sarcopenia and healthy group were significantly enriched in GnRH signaling pathway.

GO enrichment analysis showed that these 88 DEPs were significantly enriched in 112 GO terms, including response to stress, multi-organism process, transition metal ion binding, and negative regulation of multicellular organismal process (Table S2). The top 20 significantly enriched pathways in the GO term were displayed in Fig. 3A. KEGG showed that these 88 DEPs were highly enriched in the GnRH signaling pathway (Table S2; Fig. 3B).

### Identification of sarcopenia-related genes

**Figure 4:**
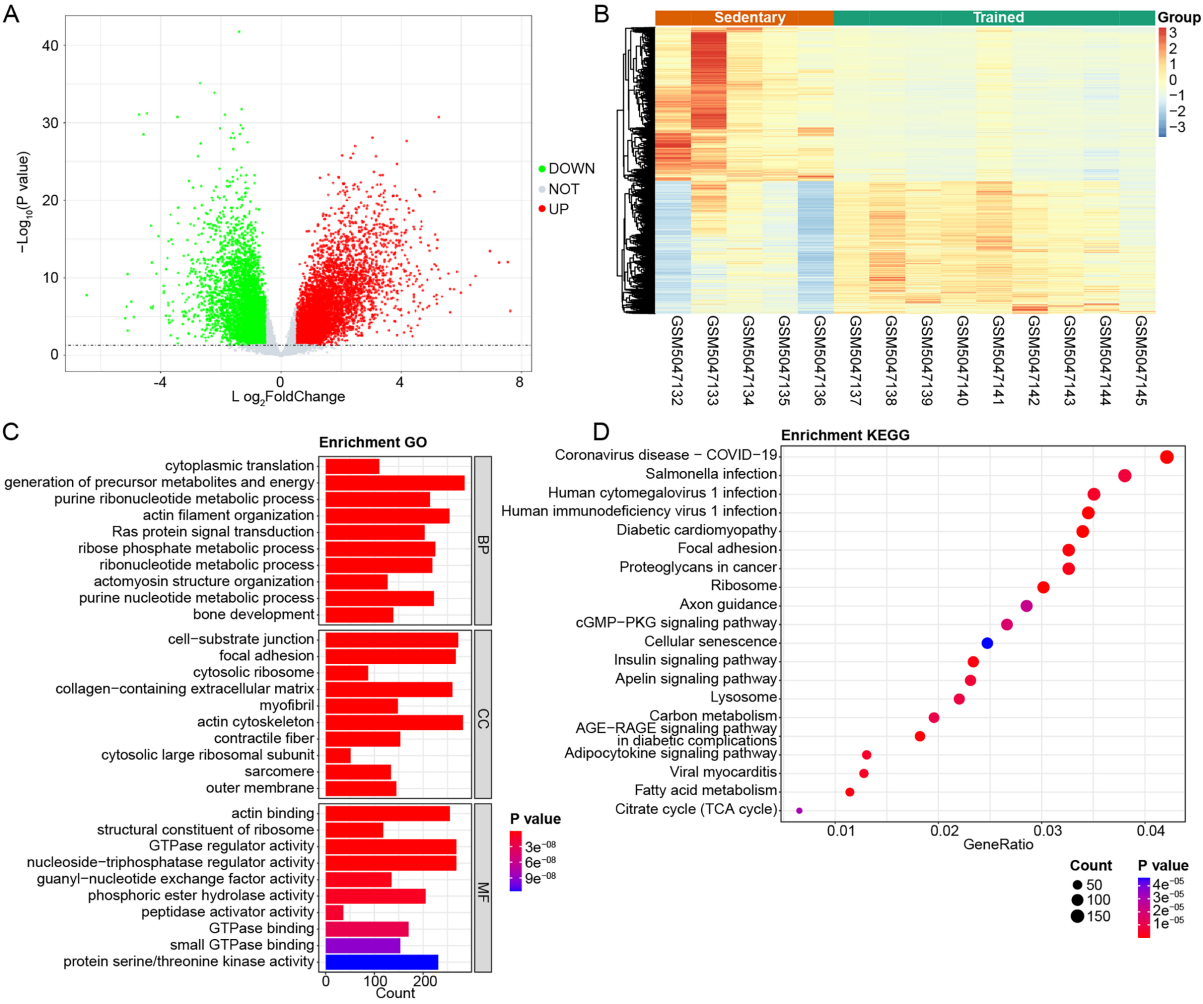
Identification of sarcopenia-related genes. (A, B) Differentially expressed genes (DEGs) between sedentary and trained samples in the SGE165630 dataset. (C) Top 30 significant results of the GO enrichment analysis. (D) Top 20 significantly enriched KEGG pathway.

To identify early sarcopenia-related genes, the transcriptome data of early sarcopenia patients were collected in the GEO database (GSE165630). A total of 9,646 DEGs were identified when comparing sedentary and trained individuals. Among these, 5,191 genes were found to be up-regulated, while 4,455 genes were down-regulated in sedentary samples compared to trained samples (Figs. 4A, 4B). Enrichment analyses showed that these 9,646 DEGs were highly enriched in 2,468 GO terms and 163 KEGG pathways, respectively (Table S3). The top 30 significant results of the GO enrichment analysis were displayed in Fig. 4C. The top 20 significantly enriched KEGG pathways are shown in Fig. 4D.

### APOD, C1QC, PVR, and MMP2 might be hub target genes in sarcopenia patients

**Figure 5:**
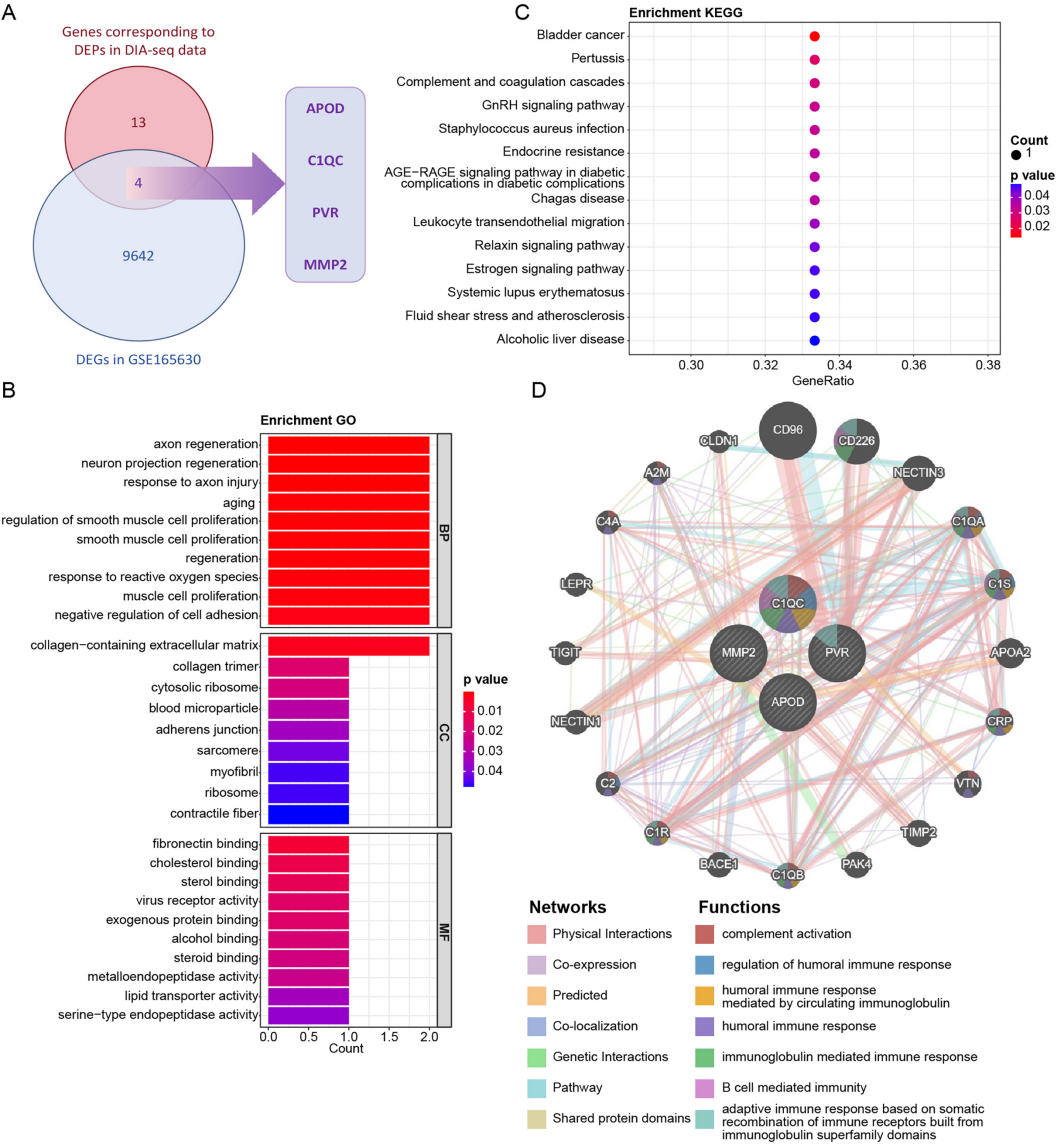
APOD, C1QC, PVR, MMP2 might be hub target genes in sarcopenia patients. (A) The result of crossover analysis between 17 coding genes and 9,646 DEGs. (B) The top 29 significant results of the GO enrichment analysis. (C) The significantly enriched KEGG pathway. (D) The interactions between these four genes and 20 genes.

To further identify hub genes related to early sarcopenia, a crossover analysis between 17 coding genes and 9,646 DEGs was performed. As a result, four overlapping genes: *APOD, C1QC, PVR*, and *MMP2* were successfully identified (Fig. 5A). Furthermore, these four genes were highly enriched in 236 GO terms, such as axon regeneration, neuron projection regeneration, and 14 KEGG pathways, including GnRH signaling pathway and complement and coagulation cascades (Table S4). The top 29 significant results of the GO enrichment analysis were displayed in Fig. 5B. The significantly enriched KEGG pathway were shown in Fig. 5C. Moreover, the interactions among *APOD, C1QC, PVR*, and *MMP2* were analyzed using GeneMANIA network, including co-localization, pathways, co-expression, and interaction patterns. As shown in Fig. 5D, there were potential interactions between these four genes and 20 genes.

### APOD was highly expressed in sarcopenia patients and had diagnostic value for sarcopenia

**Figure 6:**
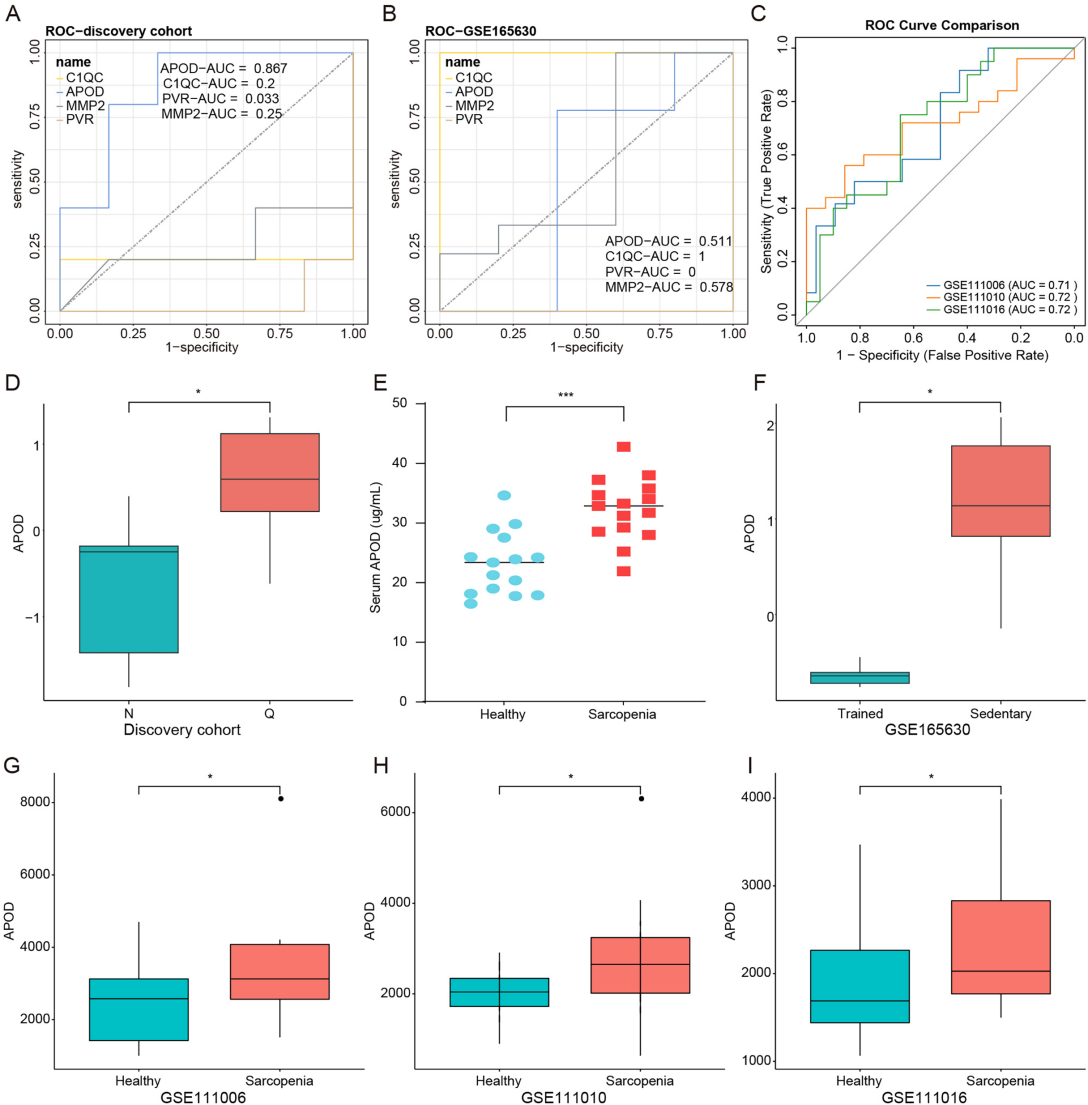
APOD was highly expressed in sarcopenia patients and had diagnostic value for sarcopenia. (A) The area under the ROC curve (AUC) for *APOD*, *C1QC*, *PVR*, *MMP2* in the discovery cohort. (B) The area under the ROC curve (AUC) for *APOD*, *C1QC*, *PVR*, *MMP2* in the GSE165630 dataset. (C) The area under the ROC curve (AUC) for *APOD* in the GSE111006, GSE111010, and GSE111016 datasets. (D) The expression of *APOD* in sarcopenia patients with low muscle mass and normal grip strength compared to healthy individuals in the discovery cohort. (E) The expression of *APOD* protein in the healthy and sarcopenia samples in the validation cohort. (F) The expression of *APOD* in sedentary samples and trained samples in the GSE165630 dataset. (G–I) The expression of *APOD* in healthy controls and sarcopenia samples in the GSE111006, GSE111010, and GSE111016 datasets. *p < 0.05, ***p < 0.001.

To investigate the diagnostic value of the four genes for early sarcopenia, receiver operating characteristic (ROC) curves were generated in the discovery cohort and the GSE165630 dataset. The results showed that in the discovery cohort, the area under the ROC curve (AUC) for *APOD* was the largest (AUC = 0.867) among four genes (Fig. 6A). In the GSE165630 dataset, the AUC for *APOD*, *C1QC*, and *MMP2* was 0.511. 1 and 0.578, respectively (Fig. 6B). However, the AUC value of APOD in the GSE111006, GSE111010, and GSE111016 datasets was greater than 0.7 (Fig. 6C). Therefore, *APOD* was selected for subsequent analysis.

In the discovery cohort *APOD* was highly expressed in early sarcopenia patients with low muscle mass and normal grip strength compared to healthy individuals (Fig. 6D). Furthermore, to independently validate this finding, serum APOD levels were measured by ELISA in a pre-planned validation cohort (15 early sarcopenia patients and 15 healthy controls). The results confirmed a significant elevation of APOD protein concentration in early sarcopenia patients compared with healthy subjects (Fig. 6E), thereby orthogonally validating the proteomic discovery. Similarly, the expression of *APOD* was higher in sedentary samples compared to trained samples in the GSE165630 dataset (Fig. 6F). In the GSE111006, GSE111010, and GSE111016 datasets, the same results were obtained (Figs. 6G–6I). Finally, the DGDB database (https://www.dgidb.org/) was used to predict drugs or molecular compounds that might interact with *APOD*, and discovered that the retinoic acid agent exhibited an interaction with *APOD*. These results indicated that *APOD* was highly expressed in early sarcopenia patients and had diagnostic value for early sarcopenia.

### Potential functional information of APOD in sarcopenia

**Figure 7:**
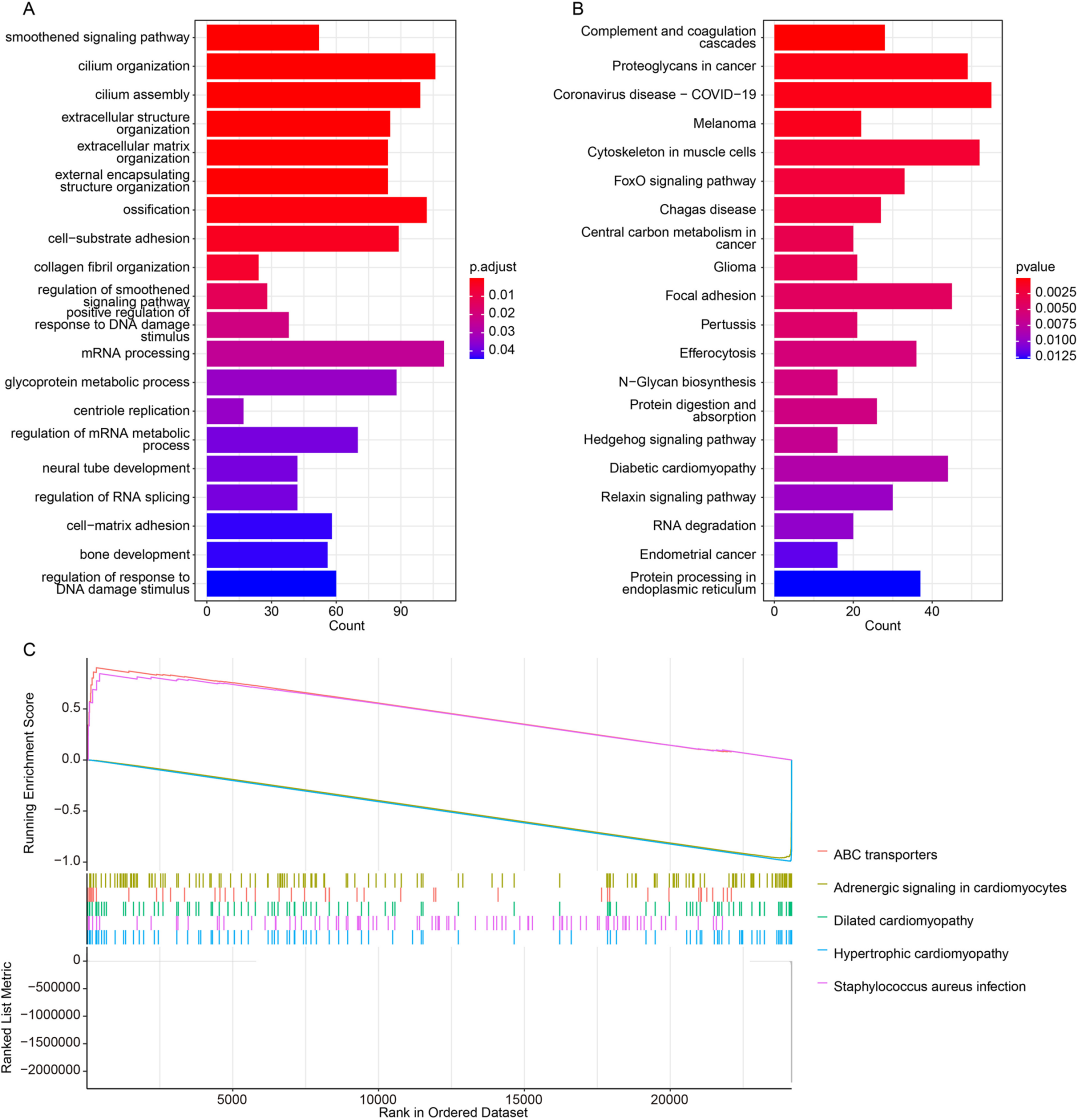
Functional enrichment analysis. (A) GO function enrichment analysis. (B) KEGG function enrichment analysis. (C) The result of gene set enrichment analysis.

In the GSE111010 dataset, early sarcopenic subjects were categorized into *APOD* high and low expression groups based on the median *APOD* gene expression level to identify DEGs. Furthermore, GO and KEGG pathway enrichment analyses were conducted based on these DEGs. The GO enrichment analysis revealed that these DEGs were significantly enriched in the smoothened signaling pathway, cilium organization, cilium assembly, extracellular structure organization, and extracellular matrix organization progression (Fig. 7A; Table S5). The KEGG enrichment analysis indicated that these DEGs were primarily enriched in complement and coagulation cascades, cytoskeleton in muscle cells, and FoxO signaling pathway (Fig. 7B, Table S6).

Furthermore, the result of gene set enrichment analysis (GSEA) showed that five signaling pathways, such as PI3K-Akt signaling pathway, Maturity onset diabetes of the young, were significantly activated in *APOD* high group, and ABC transporters, and 16 signaling pathways, such as adrenergic signaling in cardiomyocytes, Dilated cardiomyopathy, hypertrophic cardiomyopathy, were significantly inhibited in *APOD* high group (Fig. 7C, Table S7). These findings suggest that APOD may contribute to the development of early sarcopenia by modulating metabolic, stress-response, and cardiovascular-related pathways. This potential role is particularly significant because early sarcopenia is fundamentally characterized by low muscle mass in the context of incipient metabolic dysfunction.

## Discussion

The diagnosis of early sarcopenia has garnered significant clinical and research interest, with recent advancements including the application of machine learning to enhance screening accuracy (Buccheri et al., 2024). Despite these innovations, current diagnostic approaches often involve costly or specialized assessments, underscoring the continued need for accessible and reliable biomarkers to identify individuals at risk. This study integrated in-house serum proteomics with public transcriptomics to identify protein signatures in older adults with low muscle mass, a hallmark of early sarcopenia. This multi-omics approach revealed *APOD* as a promising diagnostic biomarker for the condition.

The loss of muscle strength and muscle mass were significant factors that contribute to mobility challenges among older individuals, especially leading to a decrease in walking speed (Lauretani et al., 2003). Therefore, developing diagnostic markers for sarcopenia is important for early diagnosis and intervention. Current diagnostic criteria for sarcopenia rely on a combination of measurements, such as muscle mass, muscle strength, and physical performance (Cruz-Jentoft et al., 2019a). However, a standardized set of biomarkers that can accurately identify sarcopenia is still lacking. Potential biomarkers under investigation for sarcopenia include markers of muscle inflammation (Tuttle, Thang & Maier, 2020), oxidative stress (Ozsurekci et al., 2021), and muscle protein turnover (Gumucio & Mendias, 2013). These markers can provide valuable insights into the underlying biological mechanisms of sarcopenia and aid in its diagnosis. For example, C-reactive protein (CRP), TNF-α, IL-6, IGF-1 (Tuttle, Thang & Maier, 2020), and myostatin (Yasar et al., 2022) have been associated with muscle wasting and dysfunction in various studies. However, the underlying diagnostic utility of these biomarkers in sarcopenia remains unclear. Thus, identification of reliable diagnostic markers for sarcopenia is a vital step in early diagnosis and intervention of sarcopenia. This study analyzed the characteristics of serum protein expression between early sarcopenia patients with low muscle mass and healthy individuals. It was found that a total of 88 proteins were significantly expressed between early sarcopenia patients and healthy individuals, and these 88 proteins were highly enriched in multiple biological processes, including negative regulation of the immune system process, direct ossification, and stress response. Moreover, these 88 proteins correspond to 17 coding genes, among which *APOD* had diagnostic value for early sarcopenia.

APOD is a glycosylated protein that belongs to the lipocalin superfamily of hydrophobic molecule carriers (Rassart et al., 2020). *APOD* is primarily expressed by fibroblasts, especially those near blood arteries (Rassart et al., 2020). In addition, the *APOD* gene is found to be significantly upregulated in various diseases, such as cancer (Jankovic-Karasoulos et al., 2020), neurodegenerative disease (Alzheimer, Parkinson) (Belloir et al., 2001; Ordonez et al., 2006), and hypothyroidism (Salami et al., 2019). It is important to note that the upregulation of *APOD* is not unique to sarcopenia. As highlighted by previous studies, elevated APOD protein expression across diverse pathological states often shares underlying features of metabolic dysregulation and chronic inflammation. Therefore, *APOD* is unlikely to be a disease-specific biomarker. Instead, the present findings suggest that it may serve as a shared blood-based indicator of the metabolic and inflammatory perturbations that are also central to the pathogenesis of early sarcopenia. This interpretation is supported by the bioinformatics analyses linking *APOD* to key pathways such as PI3K-Akt signaling and adipocytokine signaling. Thus, the clinical value of *APOD* in the context of early sarcopenia may lie in its ability to reflect this common dysfunctional state, aiding in the identification of individuals undergoing early muscle metabolic decline, rather than serving as a standalone diagnostic tool. Correspondingly, in the present study, *APOD* was also highly expressed in early sarcopenia patients and demonstrated diagnostic value for this condition.

The biological functions of *APOD* are mainly related to neuroprotective effects and lipid metabolism (Perdomo & Henry Dong, 2009; Ganfornina et al., 2010). In addition, it has been reported that *APOD* is correlated with inflammation and oxidative stress (Fyfe-Desmarais et al., 2023). In the present study, *APOD* might contribute to sarcopenia progression by modulating inflammatory (PI3K-Akt pathway) and metabolic (adipocytokine signaling pathway) mechanisms. Notably, lipid metabolism, inflammation, and oxidative stress significantly contribute to the pathogenesis of sarcopenia. Compared to healthy muscle, sarcopenic muscle showed altered PPARα- and ATGL-mediated lipid signaling pathway, imbalanced oxidative and nitrosative conditions, and elevated pro-inflammatory cytokines (TNF-α, IL-6) (Aquilano et al., 2016). In sarcopenia patients, the imbalance between lipolysis rate and clearance is disrupted, exacerbating lipolysis and leading to more free fatty acid (FFAs) in the blood and ectopic accumulation in skeletal muscle (Al Saedi et al., 2022). Insulin resistance is linked to sarcopenia’s primary pathogenesis (Garn & Smith, 1980). One of the main factors of insulin resistance in the skeletal muscle is increased fatty acid absorption and fatty acid intermediate buildup (Zhang et al., 2010). The disruption of lipid acid metabolism is a primary cause of insulin resistance throughout the body. Activation of serine/threonine kinases by fatty acid intermediates impairs insulin receptor activation of downstream targets, leading to decreased translocation of glucose shipment protein 4 and reduced glucose uptake by skeletal muscle cells, leading to insulin resistance (Summers et al., 1998). Moreover, insulin signaling is inhibited by fatty acid-induced inflammation and cellular lipid excess, which cause oxidative damage and endoplasmic reticulum stress (Chandrahas, Han & Kaufman, 2018). Accordingly, it is reasonable to hypothesize that *APOD* may impact the development of early sarcopenia in later stages of life by regulating lipid metabolism, inflammation, and oxidative stress pathways. However, further studies are required to substantiate this hypothesis.

While this study provides initial evidence supporting the association of *APOD* with early sarcopenia, several limitations should be acknowledged. First, the sample size of the discovery cohort was relatively small, which may limit the generalizability of the findings. Second, due to the cross-sectional nature of the study, a causal role for *APOD* in disease pathogenesis cannot be inferred, nor can its value in predicting the progression of early sarcopenia be determined. The influence of external modifiable factors on APOD expression remains to be investigated. Third, the functional insights derived from bioinformatics analyses, although informative, require further experimental validation to elucidate the precise role of *APOD* in the pathophysiology of early sarcopenia. Future studies employing larger longitudinal cohorts and mechanistic experiments are warranted to confirm the diagnostic and biological relevance of APOD in this condition.

## Conclusion

In conclusion, this cross-sectional study, employing a discovery-validation design, identified and validated serum *APOD* as a biomarker associated with early sarcopenia. Its potential immediate value lies in aiding the identification of high-risk individuals for targeted assessment. Future longitudinal studies are warranted to evaluate its predictive capacity and its responsiveness to interventions.

## Supplemental Information

10.7717/peerj.21058/supp-1 Supplemental Information 1 The coding genes corresponding to 88 DEPs.

10.7717/peerj.21058/supp-2 Supplemental Information 2 The results of 88 DEPs enriched GO term and KEGG pathway.

10.7717/peerj.21058/supp-3 Supplemental Information 3 The results of 9,646 DEGs enriched GO term and KEGG pathway.

10.7717/peerj.21058/supp-4 Supplemental Information 4 The results of *APOD, C1QC, PVR, MMP2* enriched GO term and KEGG pathway.

10.7717/peerj.21058/supp-5 Supplemental Information 5 The results of GO enrichment analysis.

10.7717/peerj.21058/supp-6 Supplemental Information 6 The results of KEGG enrichment analysis.

10.7717/peerj.21058/supp-7 Supplemental Information 7 The result of gene set enrichment analysis.

## List of abbreviations

ASM: Appendicular skeletal muscle mass
APOD: Apolipoprotein D
AWGs: Asian working group for sarcopenia
AUC: Area under the curve
DEGs: Differentially expressed genes
DEPs: Differentially expressed proteins
CRP: C-reactive protein
CV: Coefficient of variation
FFAs: Free fatty acid
GEO: Gene Expression Omnibus
iRT: Indexed retention time
KEGG: Kyoto Encyclopedia of Genes and Genomes
PPI: Protein-Protein interaction
QC: Quality control
ROC: Receiver operating characteristic
SMI: Skeletal muscle index

## Additional Information and Declarations

### Data Availability

The following information was supplied regarding data availability:

The data is available at GEO: GSE165630, GSE111006, GSE111010, and GSE111016 and iProX: IPX0004531000.

https://www.iprox.cn//page/project.html?id=IPX0004531000
